# Evaluating the psychosocial and clinical impact of diagnostic delay in psoriatic arthritis: insights from a monocentric cohort

**DOI:** 10.3389/fimmu.2026.1894497

**Published:** 2026-07-30

**Authors:** Sara Ferrigno, Francesco De Vincenzo, Marco Panetta, Maria Arena, Chiara Alessio, Michelina Turri, Lorenzo Vano, Immacolata Prevete, Felice Sensi, Anna Contardi, Gian Domenico Sebastiani

**Affiliations:** 1 Rheumatology Unit, San Camillo-Forlanini Hospital, Rome, Italy; 2 Department of Health and Life Sciences, European University of Rome, Rome, Italy

**Keywords:** diagnostic delay, early diagnosis, pain perception, psoriartic arthritis, psychosocial well being

## Abstract

**Objectives:**

Our aim was to investigate the association between diagnostic delay and the clinical and psychosocial features in patients with psoriatic arthritis (PsA), with particular focus on the mental health, wellbeing, and pain-related mechanisms.

**Methods:**

A monocentric cross-sectional study was conducted including consecutive patients with PsA classified according to the Classification Criteria for Psoriatic Arthritis. Diagnostic delay was defined as the time between symptom onset and diagnosis, and patients were stratified according to delays ≥1 year and ≥2 years. Clinical assessment included demographic and clinical parameters, such as the Psoriasis Area and Severity Index (PASI), Leeds Enthesitis Index (LEI), Disease Activity in Psoriatic Arthritis (DAPSA), and treatment history. Psychosocial evaluation included evaluation of pain, fatigue, and sleep quality using the Numeric Rating Scale (NRS) from the corresponding Psoriatic Arthritis Impact of Disease (PsAID) domains, Hospital Anxiety and Depression Scale (HADS), the Mental Health Continuum—Short Form (MHC-SF), the PainDETECT questionnaire (PDQ), the Central Sensitization Inventory (CSI), and the Brief Pain Inventory (BPI). Patients were stratified according to the presence of at least 1 year or at least 2 years of diagnostic delay. Univariable and multivariable analyses were performed to compare the demographic and clinical variables across different groups and to identify factors associated with the diagnostic delay.

**Results:**

A total of 112 patients were enrolled, with 38.4% experiencing a diagnostic delay ≥1 year and 25.8% a delay ≥2 years. Both groups showed higher BMI, longer disease duration, and higher NRS pain compared with patients with less than 1 year and less than 2 years of delay. No significant differences were observed in disease activity, fatigue, sleep, PDQ, CSI, and BPI. Lower social wellbeing assessed through the MHC Social Well-Being (MHS-SWB) subscale was observed in patients with >2 years of delay. Two logistic regression models selecting diagnostic delay >2 years as the dependent variable and MHC-SWB first, then NRS pain together with BMI, disease duration, and DAPSA as covariates, confirmed the independent association of both NRS pain (OR = 1.3, 95%CI = 1–1.6) and MHC-SWB (OR = 0.9, 95%CI = 0.8–0.9) with diagnostic delay.

**Conclusion:**

Diagnostic delay in our PsA cohort is independently associated with higher patient pain perception and reduced social wellbeing, suggesting an association with selected patient-reported outcomes beyond traditional clinical outcomes. However, the lack of association with other measures, such as central sensitization, depression, and anxiety, supports a multifactorial and incomplete interpretation, highlighting the need for larger, longitudinal studies to clarify the relationship between diagnostic delay and psychosocial outcomes.

## 1 Introduction

Psoriatic arthritis (PsA) is a chronic inflammatory disease characterized by a highly heterogeneous clinical spectrum. This complexity contributes to substantial diagnostic challenges, particularly in the early phases of the disease, when untimely identification of musculoskeletal symptoms can delay the rheumatologic referral, with recent data reporting higher diagnostic delay in PsA compared with rheumatoid arthritis (RA) (1, 2). It is well known that radiographic damage in PsA is documented within 2 years from disease onset (3, 4), although the damage can be detected even in the earlier phases of the disease. In fact, PsA patients with a delayed referral greater than 6 months demonstrated a significantly higher incidence of peripheral erosions and reduced physical function (5).

Therefore, the importance of early diagnosis and timely treatment is increasingly recognized, such as the need for early treatment and treat-to-target strategies, supported by the updated EULAR recommendations (6). However, the impact of diagnostic delay may extend beyond structural damage accrual and higher disease activity. A prolonged period of unrecognized symptoms may expose patients to persistent pain, functional limitations, and delayed access to appropriate care, potentially contributing both to physical and psychosocial burden.

There has been growing attention toward the broader burden of PsA, extending beyond articular manifestations. Patients with PsA present higher prevalence of comorbidities, such as obesity, dyslipidemia, and psychiatric conditions, compared with the general population, contributing not only to patients’ quality of life but also to their treatment response (7, 8). A substantial body of literature indicates that PsA is frequently associated with psychological distress, reduced mental wellbeing, and impaired quality of life. Systematic review data showed that PsA negatively affects emotional, social, and mental health, in addition to physical function, with pain and fatigue being the strongest correlates of a reduced quality of life (9). These comorbidities influence daily activities, work productivity, patient pain perception, and treatment response, often remaining underestimated by conventional measures of disease activity.

Depression and anxiety have been correlated with higher disease activity, mainly driven by the subjective domains, and worse physical function in different PsA cohorts (10, 11), also proving a link between depression and systemic inflammation (10).

In addition to the psoriatic disease severity, psychological distress symptoms also appear to influence treatment response. Higher anxiety and depression have been associated with a lower probability of achieving remission in PsA longitudinal cohorts, and depressive symptoms have been linked to poorer response to biologic therapy and higher discontinuation or switching rates (12, 13).

Persistent pain despite adequate control of inflammation has also raised interest in centrally mediated pain mechanisms in inflammatory arthritis. A subset of patients may experience pain that is not fully explained by peripheral inflammatory activity, suggesting the contribution of neuropathic-like pain features or central sensitization.

In PsA, neuropathic-like pain features has been associated with disease activity, but not with signs of peripheral inflammation (14). Moreover, recent data reported that patients with PsA showed higher prevalence of pain sensitivity compared with those with RA (14, 15). The prevalence of central sensitization-like symptoms was estimated to be approximately 40% in different PsA cohorts and was associated with higher disease activity, disability, and worse physical and psychological quality of life (15, 16).

Despite increasing evidence on the clinical consequences of a diagnostic delay in PsA, less is known about its association with psychosocial outcomes and pain-related mechanisms. The present study aimed to investigate whether diagnostic delay is associated with the clinical outcomes and psychosocial features in patients with PsA.

## 2 Methods

The study was designed as a cross-sectional study, which enrolled consecutive patients with PsA classified according to the Classification Criteria for Psoriatic Arthritis (17) who visited the Rheumatology Unit of the San Camillo-Forlanini Hospital from September 2024 to January 2026.

### 2.1 Demographic and clinical parameters

Data on age, sex, smoking habit, BMI, age at diagnosis, and age at symptom onset were collected. Disease duration was defined as the difference between the date of last evaluation and the date of symptom onset, while diagnostic delay was defined collecting date of diagnosis and the date of symptom onset from medical records.

Patients underwent a rheumatological evaluation, including the assessment of tender and swollen joint count and the following clinimetric scales: Psoriasis Area and Severity Index (PASI) (18) to evaluate the severity of cutaneous involvement and the Leeds Enthesitis Index (LEI) (19) and Disease Activity in Psoriatic Arthritis (DAPSA) to evaluate the entheseal and articular domain, respectively. The presence of a concomitant fibromyalgia was recorded based on a previous clinical diagnosis documented in the medical records.

Finally, ongoing treatment with prednisone (PDN) and antidepressants, together with the number of previous therapeutic lines and the number of biologic or targeted synthetic disease-modifying antirheumatic drugs (b/tsDMARDs) in a patient’s history, were reported.

### 2.2 Psychological evaluation

Patients underwent a psychological evaluation comprising psychosocial and pain-related assessments.

The Psoriatic Arthritis Impact of Disease (PsAID) is a patient-reported outcome measure developed to assess the overall impact of PsA from the patient’s perspective (20). It includes domains such as pain, fatigue, emotional wellbeing, social participation, and functional capacity and is widely used as a comprehensive measure of disease impact. Patient pain, fatigue, and sleep disturbances were assessed using the Numeric Rating Scale (NRS) derived from the PsAID questionnaire (20). Participants rate the intensity of pain over the past week on an 11-point scale ranging from 0 (no pain) to 10 (severe pain). Fatigue and sleep disturbances were assessed using the same scheme.

Depressive and anxious symptoms were assessed using the Hospital Anxiety and Depression Scale (HADS) (21), which includes two subscales, namely, HADS-A and HADS-D, designed to evaluate anxiety and depression, respectively. Each subscale consists of seven items rated on a four-point Likert scale from 0 to 3, with higher scores reflecting greater symptom severity. A cutoff value of 8 on each subscale is commonly used to distinguish individuals without clinically relevant symptoms (<8) from those with possible anxiety or depressive disorders.

Wellbeing was assessed using the Mental Health Continuum—Short Form (MHC-SF), a 14-item instrument including three dimensions: emotional wellbeing (EWB), assessed through items capturing positive affect, interest in life, and life satisfaction; psychological wellbeing (PWB), which covers domains such as self-acceptance, environmental mastery, positive relationships, personal growth, autonomy, and purpose in life; and social wellbeing (SWB), comprising items related to social contribution, integration, actualization, acceptance, and coherence. Responses are rated on a six-point scale ranging from 0 (never) to 5 (every day), yielding a total score between 0 and 70, with higher scores indicating greater overall wellbeing.

Neuropathic-like pain features were assessed using the PainDETECT Questionnaire (PDQ) (22). It includes items on pain intensity (three numeric rating scales), pain course, pain radiation (via a body diagram), and seven descriptors of somatosensory symptoms rated on a Likert scale.

A total score ranging from −1 to 38 is derived from the responses, excluding pain intensity ratings. The score incorporates pain course pattern (−1 to 1), presence of radiating pain (0–2), and severity of the somatosensory symptoms (0–35). Based on established cutoffs, scores >18 suggest a likely neuropathic component, scores <13 indicate it is unlikely, and scores between 13 and 18 are considered inconclusive.

Symptoms related to central sensitization were assessed using the short form of the Central Sensitization Inventory (CSI) (23). It consists of two parts. Part A includes nine items rated on a five-point Likert scale ranging from 0 (never) to 4 (always), with higher scores indicating greater symptoms related to central sensitization. Part B assesses the presence of previously diagnosed conditions associated with central sensitization (e.g., irritable bowel syndrome).

Pain interference was assessed using the Pain Interference subscale of the Brief Pain Inventory (BPI) (24). It consists of seven items, each assessing a specific domain: general activity, walking ability, work, relationships with others, enjoyment of life, mood, and sleep. Each item is rated on a scale from 0 (does not interfere at all) to 10 (completely interferes).

### 2.3 Statistical analysis

Quantitative data are expressed as the mean ± standard deviation, while categorical variables are expressed as number (percentage) of participants. To test the normality of the datasets, the D’Agostino and Pearson omnibus tests were used. Categorical variables were compared using the *χ*^2^ test. Continuous variables were compared using the parametric unpaired *t*-test or the non-parametric Mann–Whitney *U* test, when appropriate. *P*-values <0.05 were considered significant. Missing data were assessed before the analyses. Missing values were limited and mainly concerned occasional single items within patient-reported questionnaires. For each questionnaire, missing item-level responses were imputed using the individual mean of the completed items within the corresponding scale or subscale.

Variables included in the regression models were selected based on statistical significance in the univariate analysis and clinical relevance. Analyses were performed using IBM SPSS Statistics 26.0 for macOS.

## 3 Results

A total of 112 patients with PsA were enrolled. The demographic and clinical data of the study population are displayed in **Table 1**.

**Table 1:** Demographic and clinical characteristics of the study population.

Total no. of PsA patients, *N* = 112	
Age (years)	57.9 ± 11.3
Sex (female)	43 (38.4)
Smoking habit	25 (24)
BMI	25.9 ± 3.5
Disease duration (years)	14.4 ± 12
Diagnostic delay (years)^a^	2.4 ± 5.8
Fibromyalgia	7 (6.3)
Ongoing PDN	43 (38.4)
Ongoing antidepressants	5 (4.5)
No. of therapeutic lines	2 ± 1.3
No. of bDMARDs	0.6 ± 0.8
No. of tsDMARDs	0.2 ± 0.4
DAPSA	15.6 ± 11.9
PASI	3 ± 4.5
LEI	0.46 ± 1.2
PGA	3.9 ± 2.2
NRS Pain	4.8 ± 2.7
NRS Fatigue	5 ± 3
NRS Sleep	5 ± 3
PDQ	13.9 ± 8
MHC_tot	41.8 ± 15.2
MHC_EWB	9.5 ± 3.9
MHC_SWB	10.9 ± 6.5
MHC_PWB	21.5 ± 6.6
CSI	20.3 ± 7.7
HADS	13.2 ± 7.5
HADS—Anxiety	8 ± 4.5
HADS—Depression	5.2 ± 3.6
BPI	31.3 ± 20.2

*PsA*, psoriatic arthritis; *BMI*, body mass index; *PDN*, prednisone; *bDMARDs*, biologic disease-modifying anti-rheumatic drugs; *tsDMARDs*, targeted synthetic disease-modifying anti-rheumatic drugs; *ASDAS-CRP*, Ankylosing Spondylitis Disease Activity Score using C-Reactive Protein; *DAPSA*, Disease Activity in Psoriatic Arthritis; *PASI*, Psoriasis Area and Severity Index; *LEI*, Leeds Enthesitis Index; *PGA*, Patient Global Assessment; *NRS*, Numeric Rating Scale; *PDQ*, PainDETECT Questionnaire; *MHC*, Mental Health Continuum; *EWB*, emotional wellbeing; *SWB*, social wellbeing; *PWB*, psychological wellbeing; *CSI*, Central Sensitization Inventory; *HADS*, Hospital Anxiety and Depression Scale; *BPI*, Brief Pain Inventory.

^a^Diagnostic delay = date of diagnosis–date of symptom onset.

The mean age of the cohort was 57.9 ± 11.3 years, with 38.4% female patients. The mean disease duration was 14.4 ± 12 years, while the mean diagnostic delay was 2.4 ± 5.8 years.

According to the clinical variables, patients showed moderate disease activity, with a mean DAPSA of 15.6 ± 11.9. The mean BMI was 25.9 ± 3.5. Regarding treatments, 38.4% of the patients were receiving PDN at the time of the evaluation, and the mean number of previous therapeutic lines in our population was 2 ± 1.3. The pain, fatigue, and sleep rating scales showed a mean value of approximately 5, while the mean PDQ score was 13.9 ± 8 and the mean CSI score was 20.3 ± 7.7.

From a psychosocial perspective, the mean HADS total score was 13.2 ± 7.5. MHC and BPI indicated substantial variability across patients.

For HADS and PDQ, patients were also categorized using the validated cutoffs. There were 36 patients (32%) who reported a PDQ score >18, while 55 (49%) and 32 (28.6%) patients showed clinically relevant scores for HADS-A and HADS-D, respectively.

Stratifying patients according to the diagnostic delay revealed 43 patients (38.4%) with a delay of at least 1 year, while 29 (25.8%) showed a delay of at least 2 years (**Tables 2**, **3**).

**Table 2:** Demographic and clinical characteristics of patients stratified according to the presence/absence of diagnostic delay >1 year.

Patients variables	Diagnostic delay ≥1 year and ≥2 years: *n* = 43 (38.4%)	Diagnostic delay <1 year: *n* = 69 (61.6%)	*P*-value
Age (years)	58.7 ± 10	57.4 ± 12.1	ns
Sex (female)	14 (32.5)	29 (42)	ns
Smoking habit	9 (20.9)	16 (23.1)	ns
BMI	**26.9 ± 3.5**	**25.3 ± 3.5**	**0.02**
Disease duration (years)	**17.1 ± 12.5**	**12.6 ± 11.4**	**0.05**
Fibromyalgia	1 (2.3)	6 (8.7)	ns
Ongoing PDN	14 (32.5)	29 (42)	ns
Ongoing antidepressants	1 (2.3)	4 (5.8)	ns
No. of therapeutic lines	2.2 ± 1.4	1.9 ± 1.2	ns
No. of bDMARDs	0.7 ± 0.9	0.5 ± 0.8	ns
No. of tsDMARDs	0.3 ± 0.4	0.2 ± 0.4	ns
DAPSA	15.8 ± 10.2	15.5 ± 12.8	ns
PASI	2.8 ± 4.4	3.1 ± 4.6	ns
LEI	0.5 ± 1.2	0.4 ± 1.2	ns
PGA	3.8 ± 2	3.9 ± 2.3	ns
NRS Pain	**5.2 ± 2.6**	**4.6 ± 2.8**	**0.04**
NRS Fatigue	4.9 ± 2.8	5.1 ± 3.2	ns
NRS Sleep	4.5 ± 3.2	5.5 ± 3.4	ns
PDQ	13.1 ± 7.9	14.4 ± 8.1	ns
MHC_tot	40.9 ± 14.7	42.2 ± 15.5	ns
MHC_EWB	9.6 ± 3.9	9.4 ± 3.9	ns
MHC_SWB	9.8 ± 5.9	11.6 ± 6.7	ns
MHC_PWB	21.4 ± 6.3	21.6 ± 6.8	ns
CSI	20.1 ± 6.5	20.9 ± 8.4	ns
HADS	12.9 ± 7.3	13.4 ± 7.7	ns
HADS—Anxiety	7.8 ± 4.6	8.2 ± 4.5	ns
HADS—Depression	5.1 ± 3.3	5.2 ± 3.9	ns
BPI	30.6 ± 17.9	31.8 ± 21.6	ns

*ns*, not significant; *BMI*, body mass index; *PDN*, prednisone; *bDMARDs*, biologic disease-modifying anti-rheumatic drugs; *tsDMARDs*, targeted synthetic disease-modifying anti-rheumatic drugs; *ASDAS-CRP*, Ankylosing Spondylitis Disease Activity Score using C-Reactive Protein; *DAPSA*, Disease Activity in Psoriatic Arthritis; *PASI*, Psoriasis Area and Severity Index; *LEI*, Leeds Enthesitis Index; *PGA*, Patient Global Assessment; *NRS*, Numeric Rating Scale; *PDQ*, PainDETECT Questionnaire; *MHC*, Mental Health Continuum; *EWB*, emotional wellbeing; *SWB*, social wellbeing; *PWB*, psychological wellbeing; *CSI*, Central Sensitization Inventory; *HADS*, Hospital Anxiety and Depression Scale; *BPI*, Brief Pain Inventory. The bold text represents variables with p-values<0.05.

**Table 3:** Demographic and clinical characteristics of patients stratified according to the presence/absence of diagnostic delay >2 years.

Patients variables	Diagnostic delay ≥1 year and ≥2 years: *n* = 29 (25.8%)	Diagnostic delay <2 years: *n* = 83 (74.1%)	*P*-value
Age (years)	58.3 ± 9.1	57.7 ± 12.1	ns
Sex (female)	12 (41.3)	31 (37.7)	ns
Smoking habit	8 (27.5)	17 (20.4)	ns
BMI	**27.1 ± 3.9**	**25.5 ± 3.3**	**0.03**
Disease duration (years)	**19.1 ± 12.6**	**12.7 ± 11.4**	**0.02**
Fibromyalgia	1 (3.4)	6 (7.2)	ns
Ongoing PDN	9 (31)	34 (40.9)	ns
Ongoing antidepressants	1 (3.4)	4 (4.8)	ns
No. of therapeutic lines	**2.3 ± 1.6**	**1.9 ± 1.2**	ns
No. of bDMARDs	0.7 ± 0.9	0.6 ± 0.8	ns
No. of tsDMARDs	0.3 ± 0.5	0.2 ± 0.4	ns
DAPSA	**17.3 ± 10.6**	**15.1 ± 12.2**	ns
PASI	2.7 ± 4.3	3.1 ± 4.6	ns
LEI	0.6 ± 1.4	0.4 ± 1.2	ns
PGA	3.3 ± 1.8	4.1 ± 2.3	ns
NRS Pain	**5.8 ± 2.5**	**4.5 ± 2.8**	**0.04**
NRS Fatigue	5.2 ± 3.1	5 ± 3	ns
NRS Sleep	5 ± 3.2	5.1 ± 3.4	ns
PDQ	13.6 ± 7.5	14 ± 8.2	ns
MHC_tot	38.8 ± 15.3	42.9 ± 15	ns
MHC_EWB	9.3 ± 4.1	9.6 ± 3.9	ns
MHC_SWB	**8.4 ± 6.1**	**11.9 ± 6.4**	**0.01**
MHC_PWB	21.1 ± 6.7	21.7 ± 6.7	ns
CSI	21.5 ± 7	20.3 ± 8	ns
HADS	14.5 ± 7.4	12.8 ± 7.6	ns
HADS—Anxiety	8.7 ± 4.5	7.8 ± 4.5	ns
HADS—Depression	5.7 ± 3.5	5 ± 3.7	ns
BPI	32.8 ± 18.4	30.8 ± 20.8	ns

*ns*, not significant; *BMI*, body mass index; *PDN*, prednisone; *bDMARDs*, biologic disease-modifying anti-rheumatic drugs; *tsDMARDs*, targeted synthetic disease-modifying anti-rheumatic drugs; *ASDAS-CRP*, Ankylosing Spondylitis Disease Activity Score using C-Reactive Protein; *DAPSA*, Disease Activity in Psoriatic Arthritis; *PASI*, Psoriasis Area and Severity Index; *LEI*, Leeds Enthesitis Index; *PGA*, Patient Global Assessment; *NRS*, Numeric Rating Scale; *PDQ*, PainDETECT Questionnaire; *MHC*, Mental Health Continuum; *EWB*, emotional wellbeing; *SWB*, social wellbeing; *PWB*, psychological wellbeing; *CSI*, Central Sensitization Inventory; *HADS*, Hospital Anxiety and Depression Scale; *BPI*, Brief Pain Inventory. The bold text represents variables with p-values<0.05.

No statistically significant differences were observed between groups in terms of age, sex distribution, smoking habit, disease activity indices, or treatment-related variables. However, the group with ≥1 year and ≥2 of delay reported higher mean number of previous treatment lines, as expected, even if the result did not show statistical significance (**Table 3**).

A significantly higher BMI, longer disease duration, and higher NRS pain were reported in both groups of patients with >1 and >2 years of diagnostic delay, with more significant results in the second group.

No statistically significant differences were observed in pain frequency, NRS fatigue, NRS sleep, PDQ, CSI, and BPI.

In addition, in the group with >2 years of delay, a significant difference emerged in the social wellbeing domain, with a lower mean MHC-SWB score compared with patients with diagnostic delay less than 2 years (**Table 3**).

Two logistic regression models were performed with diagnostic delay >2 years as the dependent variable. The first model included BMI, disease duration, NRS pain, and DAPSA as covariates, while the second model included BMI, disease duration, MHC-SWB, and DAPSA. The choice of covariates was based on statistical significance and clinical relevance in order to test the independent association of NRS pain and MHC-SWB with diagnostic delay. In the first model, only NRS pain remained independently associated with diagnostic delay, while in the second model both BMI and MHC-SWB showed an independent association with diagnostic delay (**Table 4**).

**Table 4:** Logistic regression models including diagnostic delay ≥2 years as a dependent variable and the following covariates: BMI, disease duration, NRS pain, and DAPSA in model 1 and BMI, disease duration, MHC-SWB, and DAPSA in model 2.

	OR	95%CI LB	95%CI UB	*p*-value
Model 1				
BMI	1.12	0.97	1.3	ns
Disease duration	1.03	0.99	1.1	ns
NRS pain	1.3	1	1.6	0.05
DAPSA	0.9	0.9	1	ns
Model 2				
BMI	1.17	1	1.3	0.04
Disease duration	1.02	0.9	1.1	ns
MHC-SWB	0.8	0.8	0.9	0.01
DAPSA	1.01	0.9	1	ns

A *p*-value >0.05 was considered significant.

*OR*, odds ratio; *CI*, confidence interval; *LB*, lower bound; *UB*, upper bound; *BMI*, body mass index; *NRS*, Numeric Rating Scale; *DAPSA*, Disease Activity in Psoriatic Arthritis; *MHC*, Mental Health Continuum; *SWB*, social wellbeing; *ns*, not significant.

## 4 Discussion

In this monocentric, cross-sectional study, we investigated whether diagnostic delay in patients with PsA is associated with a distinct clinical and psychosocial profile. To the best of our knowledge, this is among the first studies evaluating mental health, psychological distress, neuropathic-like pain features, and central sensitization-related symptoms in relation to diagnostic delay in a PsA cohort.

Overall, our cohort showed a relevant burden of patient-reported symptoms. Almost one-third of patients had PDQ scores suggestive of likely neuropathic-like pain features and HADS depression scores compatible with possible depressive symptoms. Interestingly, half of our sample reached the clinically significant values for the HADS—Anxiety subscale, indicating a high prevalence of possible anxiety symptoms.

The main finding of the present study was that patient pain and the social wellbeing domain of MHC-SF showed an independent association with a diagnostic delay greater than 2 years.

While the use of MHC-SF in PsA has been limited, a previous study in patients with RA showed higher mean values compared with those observed in our cohort (25). Although this may suggest a relevant psychosocial burden in PsA compared with other inflammatory arthritis, future studies are needed to directly compare these populations.

In the present study, lower social wellbeing was associated with diagnostic delay >2 years. According to Keyes’ conceptualization, social wellbeing reflects public and social aspects of functioning and includes five dimensions: social coherence, social actualization, social integration, social acceptance, and social contribution (26). Individuals are considered to function well socially when they perceive society as meaningful and understandable, see society as having potential for growth, feel that they belong to and are accepted by their communities, accept others, and perceive themselves as contributing to society (26). Therefore, in the context of PsA, lower MHC-SWB scores may reflect a reduced sense of belonging, social acceptance, and perceived contribution rather than only a reduced participation in social activities.

This distinction is relevant when comparing our findings with those of a previous study by Zundell et al. (27), who reported that diagnostic delay >6 months in a large survey-based PsA cohort was associated with a higher likelihood of depression, a reduced ability to participate in social roles and activities, and poorer quality of life. Our findings are partially consistent with that study as a longer diagnostic delay was also associated with a poorer social dimension. However, while Zundell et al. (27) focused on functional limitations in social participation, our results indicate an association between longer diagnostic delay and broader subjective and evaluative dimensions of social functioning, including how patients perceive their place and role within the social environment.

Unlike Zundell et al. (27), we did not observe significant associations between diagnostic delay and anxiety or depressive symptoms. Importantly, our study extends previous evidence by including the evaluation of additional pain-related domains, showing that diagnostic delay was not associated with neuropathic-like pain features, central sensitization-related symptoms, or pain interference. Overall, these findings may suggest a selective pattern of association between diagnostic delay and patient-reported outcomes in PsA, involving pain perception and social wellbeing rather than a generalized increase across all psychosocial and pain-related domains. However, these results should be considered exploratory given the cross-sectional design and the limited sample size.

Our findings also showed that delays of ≥1 and ≥2 years are both associated with higher BMI. Although the time between diagnosis and BMI assessment was variable across our cohort, this observation is further supported by previous literature findings showing a relationship between higher BMI and an increased probability of a delayed diagnosis in PsA (28). Obesity is also associated with higher disease activity, worse outcomes, and altered pain perception, potentially contributing to increased long-term disease burden (29).

Finally, our results do not support a strong association between diagnostic delay and later psychosocial or centrally driven pain-related outcomes. The lack of significant differences in PDQ and CSI suggests that neuropathic and central sensitization pain features do not clearly correlate with the time of diagnosis, in line with the multifactorial nature of pain in PsA.

This study has several limitations. The cross-sectional design does not allow confirming temporal or causal associations. In fact, the psychosocial and clinical variables were assessed at a single time point after disease onset and diagnosis; therefore, it was not possible to infer a temporal dynamic between the development of these features and the diagnostic delay. Moreover, given the exploratory nature of the study, no formal adjustments for multiple testing were applied in the univariate analysis, increasing the risk of type I error. The limited sample size also prevented us from including additional potential confounders in the multivariable regression models.

In addition, symptom onset and diagnosis were retrospectively collected and, for the majority of records, were available only with yearly granularity. Therefore, a 6-month diagnostic delay threshold could not be reliably evaluated. Future longitudinal studies are needed to determine whether earlier delay thresholds show similar associations with clinical, psychosocial, and pain-related outcomes in PsA.

Fibromyalgia was based on clinically documented diagnoses and was not systematically assessed at study inclusion, and this could explain the lower prevalence reported in our cohort compared with previous studies (30). Therefore, the low frequency observed in our cohort may have limited our ability to detect a potential association between fibromyalgia and diagnostic delay. Overall, our results should be interpreted with caution and considered as exploratory. Future multicentric, longitudinal studies are needed to validate these results in PsA.

In conclusion, in our cohort, diagnostic delay was associated with higher patient pain perception and reduced social wellbeing, suggesting that diagnostic delay may be associated with broader patient-reported outcomes beyond traditional clinical outcomes. However, no association was found with psychological, neuropathic-like pain features and central sensitization symptoms, suggesting a more multifactorial genesis of these conditions. Finally, these findings further emphasize the importance of early diagnosis and a patient-centered approach in PsA management, including the assessment of pain and mental health and the need for larger, longitudinal studies to better explore the relationship between diagnostic delay and psychosocial outcomes.

## Data Availability

The raw data supporting the conclusions of this article will be made available by the authors, without undue reservation.
